# Update of the pediatric hypertension graphic adjusted for gender and height percentiles: systolic blood pressure for girls, 1 to 17 years old

**DOI:** 10.1186/cc12635

**Published:** 2013-06-19

**Authors:** HH Shieh, AE Gilio, VHK Koch, DC Raulik, C Vranjac, S Fukugava, ER Barreira, EJ Troster

**Affiliations:** 1 University Hospital of Universidade de São Paulo, Vila Iara, São Paulo, SP, Brazil

## Introduction

Hypertension is the most important preventable risk factor for premature death worldwide. It increases the risk of ischemic heart disease, strokes, peripheral vascular disease, and other cardiovascular diseases, including heart failure, aortic aneurysms, diffuse atherosclerosis, and pulmonary embolism. In childhood, hypertension can be determined according to a table adjusted for height, age and gender [1]. A graphic representation of pediatric hypertension was published in 1987 [2], and no graphic updates have been published since then. The objective of this study was to update the graphic representation of pediatric hypertension.

## Methods

We used a computerized calculation method to develop high-resolution graphics containing curves with 5,841 points each, to depict the main percentiles associated with high blood pressure for girls from 1 to 17 years old in the 50th percentile of height. Each point represents the calculation of the polynomial equation that includes the statistical processing of the last Report on Blood Pressure [1]. We also analyzed the effect of height on blood pressure in the 5th to 95th percentile range. Statistical functions generated by computerized program were used.

## Results

**Figure 1:**
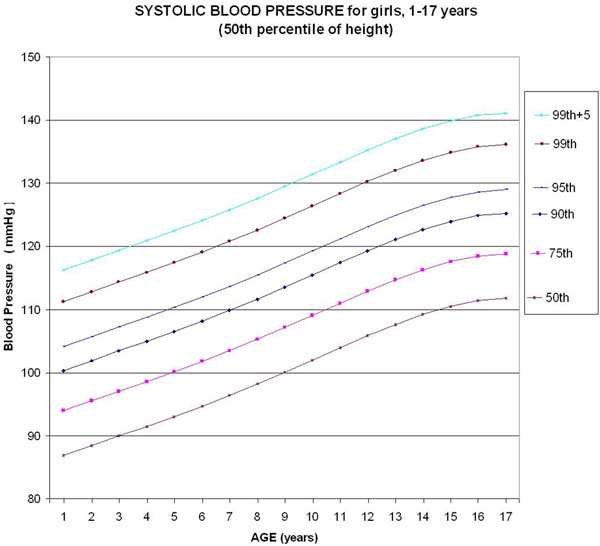
**Update of chart for systolic blood pressure (SBP) based on the last Report on Blood Pressure in 2004 **[1]**, for girls 1 to 17 years old (50th percentile of height)**. Considering a tolerance of 1 mmHg, the curve of adjustment for height of the SBP for girls does not need any correction in the in the 31.5th to 68.5th percentile of height.

Six monotonic curves of systolic BP for girls representing the 50th, 75th, 90th, 95th, 99th, and 99th +5 percentiles were built (Figure 1). In relation to the table published by the NIH, we confirm use of approximation of the values (rounding) in the published table by truncation. Considering a tolerance of 1 mmHg, the monotonic curve of adjustment for height of the SBP for girls does not need any correction in the 31.5th to 68.5th percentile of height, but needs maximal correction for the 5th percentile for height (-4.7 mmHg correction).

## Conclusion

The correction of systolic BP for height is of little significance, and this updated graphic can be used to diagnose high systolic blood pressure for girls. Clinical studies are necessary to determine the systolic BP percentile that better represents clinically significant hypertension.
